# Edge contacts accelerate the response of MoS_2_ photodetectors†

**DOI:** 10.1039/d3na00223c

**Published:** 2023-06-05

**Authors:** Fabian Strauß, Christine Schedel, Marcus Scheele

**Affiliations:** a Institute of Physical and Theoretical Chemistry, University of Tübingen Auf der Morgenstelle 18 72076 Tübingen Germany marcus.scheele@uni-tuebingen.de; b LISA+, University of Tübingen Auf der Morgenstelle 15 72076 Tübingen Germany

## Abstract

We use a facile plasma etching process to define contacts with an embedded edge geometry for multilayer MoS_2_ photodetectors. Compared to the conventional top contact geometry, the detector response time is accelerated by more than an order of magnitude by this action. We attribute this improvement to the higher in-plane mobility and direct contacting of the individual MoS_2_ layers in the edge geometry. With this method, we demonstrate electrical 3 dB bandwidths of up to 18 MHz which is one of the highest values reported for pure MoS_2_ photodetectors. We anticipate that this approach should also be applicable to other layered materials, guiding a way to faster next-generation photodetectors.

## Abstract 2

Edge contacts are compared to top contacts in terms of the photocurrent response of MoS_2_ multilayers. The faster response of the edge contacts is attributed to the predominantly in-plane direction of charge carrier diffusion.

## Graphical abstract

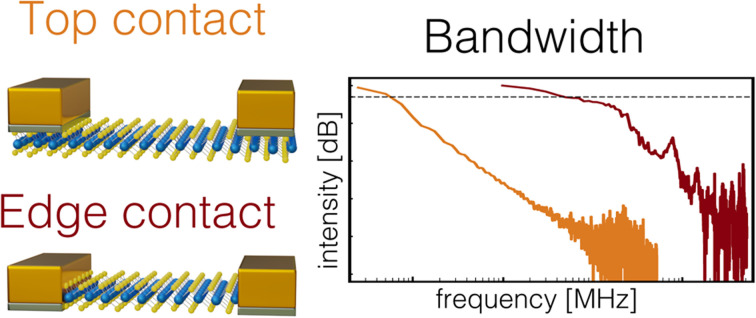

## Introduction

The rise of 2D materials in optoelectronics has led to remarkable research results in recent years, from ultra-thin transistors^1,2^ to single-layer light-emitting diodes^3^ and novel photodetectors.^4,5^ For the latter, two properties are of particular interest: device responsivity and device speed. For high frequency applications, silicon and InGaAs are the most advanced materials today,^6^ but they are starting to reach physical limits. A possible alternative is found in layered materials. The intrinsic photoresponse of these layered materials such as graphene or transition metal dichalcogenides (TMDCs) has revealed response times in the picosecond regime.^7^ This intrinsic response is a measure for the pure, material-specific photoresponse and defines an upper limit for an ideal photodetector, in which the speed is solely limited by the active material. However, in most devices the actual speed is furthermore affected by extrinsic properties related to the device geometry, such as the RC- or transit time. Pure TMDC devices exhibit typical response times ranging from milliseconds^8,9^ to seconds,^8–10^ with a few fast devices in the microsecond range^7–9,11,12^ and even less in the sub-microsecond regime.^13^ Many methods have been used in the search for ultrafast photodetectors based on nanomaterials: chemical doping,^14^ heterojunction implementation,^15,16^ photonic waveguide integration,^17^ and small channel lengths,^18,19^ to name a few. In addition, parameters such as the choice of substrate,^18,20^ interface roughness,^21^ electrode material,^10,22,23^ and geometry^13,24,25^ or environment^26,27^ must be considered each time.

**Fig. 1:**
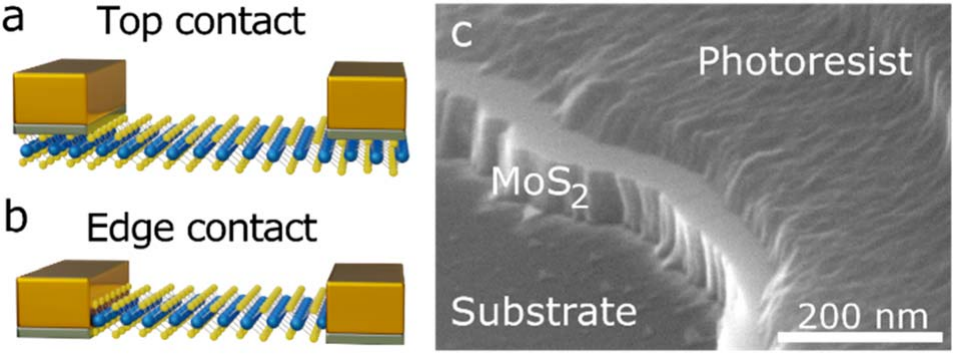
Schematic of an (a) top contacted and (b) edge contacted MoS_2_ crystal. (c) SEM image of an etched MoS_2_ flake.

Traditionally, top contacts above, or bottom contacts below the active material are used for nanomaterial photodetectors. More advanced structures can also be built by a vertical arrangement of electrodes and desired material.^13,19^ In addition, layered materials can be contacted at the edge of the flake.^28,29^ Thereby, instead of touching the surface of the layered material, *cf.*Fig. 1a, the edge of a flake is uncovered, for example with etching,^29,30^ and the electrodes are evaporated on the edge of the material, *cf.*Fig. 1b.

An SEM-image of an etched flake showing the exposed edge of a MoS_2_ flake can be seen in Fig. 1c and SI2.† These one-dimensional edge contacts have attracted much attention in recent years. They are being investigated for their reduced transfer length,^31,32^ lower contact resistance,^28,33–35^ better control of the Schottky barrier^36,37^ higher capability of charge injection^37,38^ or Fermi level depinning,^28,30,36^ to name a few examples. However, to our knowledge, the effect of edge contacts on the response time properties of TMDC photodetectors is still unknown.

In this work, we investigate the difference between edge electrodes and conventional top electrodes and their influence on the response speed of pure MoS_2_ photodetectors. The MoS_2_ flakes studied here were exfoliated using a standard scotch tape/PDMS exfoliation method.^29^ They are between 50 and 200 nm thick, see Fig. SI1e,† and can thus be considered bulk crystals where the height no longer affects the properties.^12^

We show that the electrode geometry of the device – edge electrodes instead of top contacts – accelerate the decay time by at least an order of magnitude. Primarily, we have studied the steady state photoresponse towards a 635 nm square pulse with some additional non-steady state measurements towards a 636 nm impulse laser. By using these techniques, pure MoS_2_ devices can be realized with one of the highest recorded bandwidths of more than 18 MHz. We hypothesize that this geometry should be particularly advantageous for TMDCs in view of the much more efficient in-plane transport compared to transport across several van-der-Waals layers.

## Experimental section

### Fabrication

MoS_2_ detectors were fabricated by exfoliating TMDC flakes with scotch tape onto cleaned glass substrates functionalized with HMDS. For the bottom contacts, the flakes were stamped directly onto lithographically fabricated electrodes (2.5–20 × 80 μm). For all other electrode geometries, optical lithography was performed directly on the flakes using a maskless aligner (μMLA Heidelberg Instruments). For the edge contacts, an additional plasma etching step is performed before vapor deposition of the electrode material. The platelets were etched with a mixture of O_2_/SF_6_ plasma (100 W, 25% O_2_/75% SF_6_, 50 mTorr, 20 s). The electrode material, *e.g.*, 4 nm Ti and 20 nm Au, was evaporated at a pressure of <2 × 10^−6^ mbar. The detectors were examined under atmospheric conditions.

For the Ti : Au top contacts as well as the Au edge electrodes, 4 nm Ti and 20 nm Au were evaporated. For the Ti edge contacts, 20 nm Ti and 4 nm Au or only 25 nm Ti were evaporated. Since they have shown similar behavior, their data is shown jointly. The Au bottom contacts are made of 4 nm Ti and 20 nm Au and the Ti bottom contacts of 25 nm Ti.

### Transient photoresponse

Time-resolved photocurrent measurements were performed at room temperature in a Lake Shore Cryotronics CRX-6.5K probe station. Square pulse illumination of the photodetectors was used to measure the steady state photoelectric response. For this, a fast-switching laser driver (FSL500, PicoQuant), driven by a Hewlett Packard 33120A arbitrary waveform generator was used. The 635 nm laser diode has an optical output power of ≤12 mW and a laser rise time of <0.5 ns. The non-steady state photoresponse of the detectors was investigated with a picosecond pulsed laser driver (Taiko PDL M1, PicoQuant) utilizing a 636 nm laser head with a pulse width <500 ps. For a repetition rate of 100 kHz, an average optical power of 22 μW was chosen. The laser powers were further reduced due to decollimation of the beam, inefficient coupling into the optical fiber and scattering. An unfocused beam was used with a laser spot larger than the detector area.

50 Ω matched tungsten probes and 40 GHz coaxial cables as short as possible were used to contact the detector with the external circuit. A transimpedance amplifier (FEMTO DHPCA-100) was used to preamplify the current before measuring with a Zurich Instruments UHFLI lock-in amplifier with a Periodic Waveform Analyzer function averaging the signal from 2 G samples. Before further analysis, the signals were background corrected. The bandwidth of the lock-in amplifier is 600 MHz which is further reduced to 14–200 MHz by the variable gain transimpedance amplifier.

For some contacts, the Fourier transform of 100 kHz measurements was performed after applying zero padding to mimic a 25 kHz measurement and determine the 3 dB bandwidth.

## Results and discussion

### Steady state photoresponse of edge and top contacted MoS_2_

Square pulse laser measurements determine the rise time to reach steady state as well as the corresponding fall time. This allows investigating the influence of changing the electrode geometry and/or the contacting metal for both parameters, which we used to study the effect of an edge contact compared to titanium : gold top electrodes (Ti : Au top). For further information and a scheme of the measurement setup used, the interested reader is referred to our previous work.^18^

**Fig. 2:**
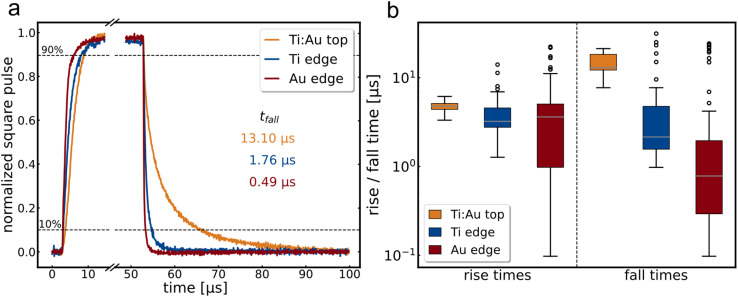
(a) Normalized square pulse response of a top contact (ochre), a titanium edge contact (blue) and a gold edge contact (red). All measurements performed with a 635 nm square pulse laser with 10 kHz repetition rate, 15 μm channel length, 0.1 V bias applied. (b) Box-and-whisker-plots of all 10 kHz, 635 nm square pulse measurements for each electrode configuration. The left half displays the rise times, and the right half the fall times. Measurements of 18 Ti : Au top, 74 Au edge, 46 Ti edge measurements contacts are included at various bias voltages between −1 and 1 V.

The edge geometry is either fabricated with the same ratio of Ti : Au (Au edge) or with the inverted ratio of titanium and gold (Ti edge), referring to the predominant contacting metal. See Fig. SI3† for the fabrication process. Typical square pulse responses of these three geometries are shown in Fig. 2a.

The fall time accelerates tremendously for the edge geometry, while the rise time accelerates only slightly. We attribute this result predominantly to the geometry but note that the metal also plays an important role. The significance of each effect can be seen in Fig. 2b, which shows the box-and-whisker plots across all 10 kHz square pulse measurements for all investigated Ti : Au top and Ti and Au edge contact flakes (4 Ti : Au top, 8 Au edge, 5 Ti edge contacts; 18 Ti : Au top, 74 Au edge, 46 Ti edge measurements). Thereby, the box includes all values from the first to the third quartile, while the whiskers span to the value maximal 1.5 times the box size away. Every value exceeding this range is displayed as an outlier. Again, the rise time does not show much improvement in the median values, although some contacts display much faster rise times for the edge contact compared to the top contacts. In contrast, the fall time shows a massive acceleration for the edge contacts, an improvement of at least a factor of eleven for the mean value compared to the top contacts. This uneven acceleration can be attributed to the multiple mechanisms that play a role in the rise time compared to the fall time, like generation of electron–hole pairs. The large spread in the Au edge data is caused by the slightly bigger sample size, a pronounced persistent photocurrent effect for especially higher bias voltages,^39^*cf.* Fig. SI4,† and the specific challenges of the fabrication of edge contacts: as can be seen in Fig. 1c, the etched flank is bolt upright, but it has a slight tendency to be tilted either inwards or outwards, thereby changing the contact angle for the edge electrodes and thus the quality of the contact. To further improve the edge contact in the future, more dedicated procedures like the etching process by Cheng *et al.*^31^ may be beneficial, which has the benefit to produce edge contacts with greater reliability and control over the environment.

We attribute the accelerated response times to the improved contacting method. Conventional top contacts with vapor deposited electrodes have many defects and hybridization in the first layers,^40^ eliminating the van der Waals gap between the electrode and TMDC. Underneath, however, are many more layers with vdW gaps and thus tunnelling barriers that must be overcome to inject a charge carrier into the electrode. In the geometry of the edge electrodes, (nearly) all layers are contacted and charge transport to the contact occurs in the plane of the MoS_2_ crystal, which has much higher mobilities.^41^ This greatly enhances the carrier injection.^28^ Moreover, the edge contacts may form covalent bonds with the dangling bonds of the TMDCs,^28^ which further improve charge transport.

**Fig. 3:**
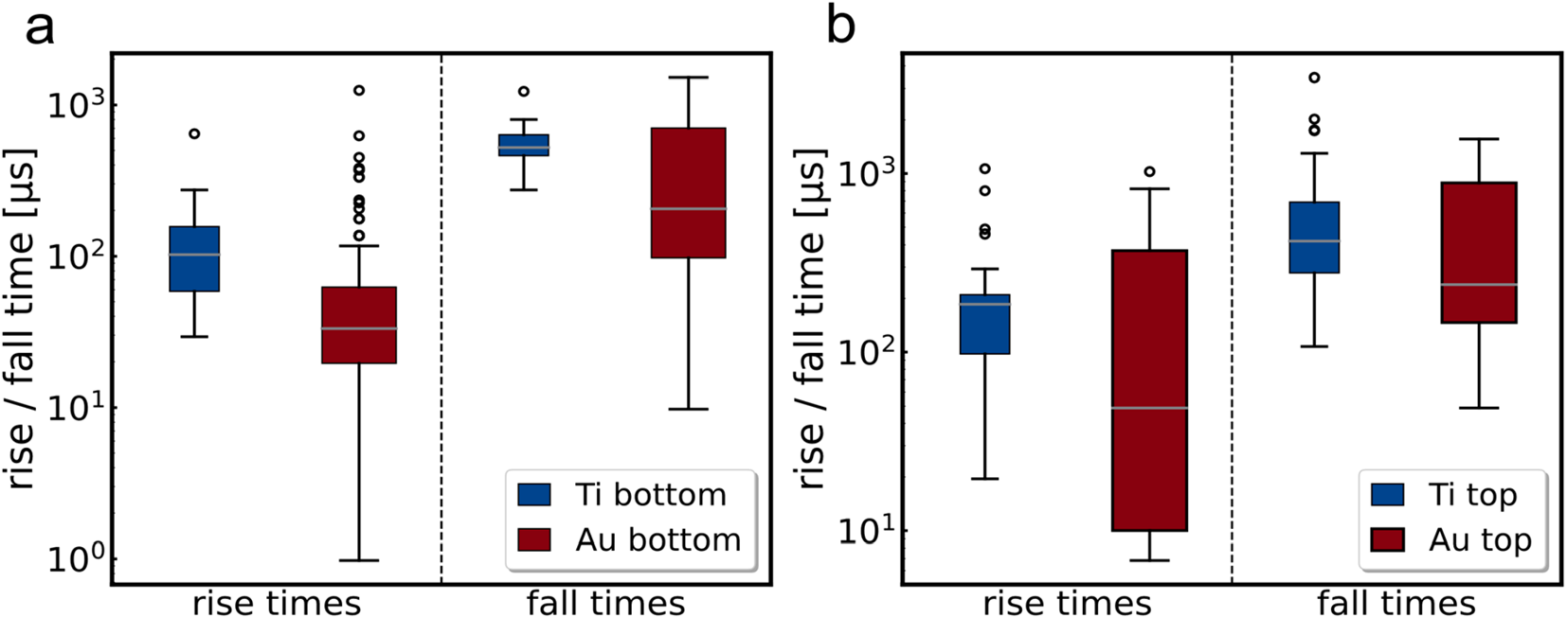
Box-and-whisker plots of rise and fall times of MoS_2_ flakes contacted with either titanium (blue) or gold (red). (a) Bottom electrode geometry, (b) top electrodes where the different channels on the same flake are contacted with titanium or gold. All measurements are carried-out with a 635 nm laser and a repetition rate of 100 Hz.

In addition to the different electrode geometry, the contacting metal has also been shown to have an impact on device speed. To quantify this influence, MoS_2_ flakes were investigated in a bottom electrode geometry with either titanium or gold as contact material (see Fig. 3a).

In addition, an analysis was performed by patterning the same flake twice so that some channels were contacted with the Ti : Au top electrodes and others with Au top electrodes only (see Fig. 3b). Both sets of experiments show the same trend already observed in the edge contact experiments, namely an acceleration of the rise and fall times with gold as the contact metal. However, the mean value of all Ti edge contacts is still faster than the best top contacts, demonstrating that the contact geometry remains an important factor for the overall response speed of the MoS_2_ photodetectors.

The influence of the contacting metal on the electrical or optoelectrical behaviour of materials is not new and has been detailed for instance in the work of Jain *et al.*^42^ Nevertheless, it is often discussed only in terms of contact resistance or dark currents, *cf.* Fig. SI5,† but rarely in terms of response times. For TMDCs, a study by Zhang *et al.*^22^ already investigated the rise time effects of palladium and titanium contacts on WSe_2_. The authors explained the faster response for titanium with a higher Schottky barrier. In the case of Au, Ti, and MoS_2_, the respective work functions are about 5.4 eV,^43^ 4.3 eV,^44^ and^44^ 3.9 eV (ref. 45) for the edge facet of MoS_2_ or 5.4 eV (ref. 46) for the top facet. Thus, regarding the titanium gold comparison, the higher Schottky barrier is expected for Au contacts, which are shown to be faster in the experiments.^38^ Furthermore, in a comparison of Au with the corresponding work functions of MoS_2_, again, the higher Schottky barrier is found for the faster contact geometry – edge contacts, *cf.* Fig. SI6.† A detailed analysis of the work functions in the different contact geometries shown here is beyond the scope of this work and would require surface tunnelling spectroscopy or similar methods.

With respect to the limiting mechanism of edge and top contact we performed measurements at different voltages which could provide hints towards a transit time limitation. Additional measurements with different channel lengths in the range from 2.5 μm to 20 μm could give further information on either a transit time limited mechanism or an RC-time limit in case of no voltage dependency. Both, voltage, and channel length variation do not show any trend, *cf.* Fig. SI7 and SI8,† thereby a limiting mechanism cannot be determined. Further distinctions towards an RC limitation would require impedance measurements.

In summary, MoS_2_ detectors can be accelerated enormously by using edge contacts in comparison to top contacts, *cf.* Ti : Au top and Ti edge in Fig. 2a. An additional tuning by using an appropriate electrode metal as already announced in literature can further help to accelerate the detector especially in the fall times, as shown by using Au edge electrodes.

### Non-steady state photoresponse of edge and top contacted MoS_2_

The investigation with a delta-shaped laser pulse mimics the data transmission in optical fibres and is therefore of relevance to determine the bandwidth of the device. For this purpose, the impulse response (*f*(*t*)) must be Fourier transformed (FFT) to obtain the power spectrum (*P*(*ω*)): *P*(*ω*) = |FFT(*f*(*t*))|^2^ After normalization with the steady-state power (*P*_1_), the bandwidth spectrum can be converted to dB = 10 log_10_(*P*(*ω*)/*P*_1_). The 3 dB bandwidth is then a measure of the frequency at which the power of the signal drops to half its value.

**Fig. 4:**
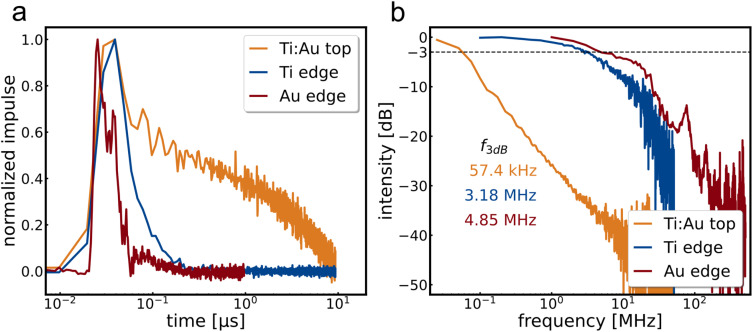
(a) Normalized impulse photoresponse of a Ti : Au top (ochre), a titanium edge (blue) and a gold edge (red) contacted MoS_2_ device towards a 636 nm pulsed laser. Measured are 10 μm contacts under a 1 V bias with either 100 kHz for the Ti edge and Ti : Au top device or 1 MHz for the Au edge device. (b) Fourier transformed impulse response to determine the 3 dB bandwidth of the device. For the top contact zero-padding is performed to mimic a quasi 25 kHz measurement.

Most flakes have relatively little signal, so it is not possible to determine the impulse photoresponse for each bias voltage and channel. Thus, no further information about the limiting mechanism can be obtained, only a representative trend, which can be seen in Fig. 4.

Fig. 4a shows exemplary impulse responses of the three types of detectors investigated and Fig. 4b the corresponding bandwidth spectra obtained by fast Fourier transform. As with the steady-state measurements, the trend shows the acceleration of the bandwidth when using edge contacts by *a* factor of more than 80 times for the example shown. The fastest device response exceeding a bandwidth of 18 MHz can be seen in Fig. SI9.†

## Conclusions

In summary, we have demonstrated the accelerating effect of edge contacts for the response of MoS_2_ photodetectors compared to the conventionally used top contacts. This improvement is caused by the better charge carrier injection into the electrodes due to the higher in-plane mobility compared to the out-of-plane mobility of TMDCs. In addition, the contacting metal and the associated Schottky barrier play an important role: for MoS_2_, gold leads to faster rise and fall times than titanium. By combining these two effects, we have built photodetectors with a bandwidth as high as 18 MHz, which, to the best of our knowledge, surpasses all neat MoS_2_ photodetectors developed so far. We believe that the implementation of edge contacts in photodetectors made of 2D materials has great potential due to their scalability and simplicity.

## Conflicts of interest

There are no conflicts to declare.

## Supplementary Material

NA-005-D3NA00223C-s001
