# Supplementary material for: Edge contacts accelerate the response of MoS2 photodetectors
Source: Nanoscale Adv. 2023 Jun 5;5(13):3494–9. doi: 10.1039/d3na00223c (PMC10295078; doi:10.1039/d3na00223c)
Supplement: NA-005-D3NA00223C-s001 [file NA-005-D3NA00223C-s001.pdf]

Electronic Supplementary Material (ESI) for Nanoscale Advances.
This journal is © The Royal Society of Chemistry 2023

# SUPPLEMENTARY INFORMATION

# Edge Contacts accelerate the Response of $MoS_2$ Photodetectors

*Fabian Strauß[1,2], Christine Schedel[1], Marcus Scheele[1,2,*]*

[1] Institute of Physical and Theoretical Chemistry, University of Tübingen, Auf der Morgenstelle 18, 72076 Tübingen, Germany

[2] LISA+, University of Tübingen, Auf der Morgenstelle 15, 72076 Tübingen, Germany

## Light microscopy images and flake height

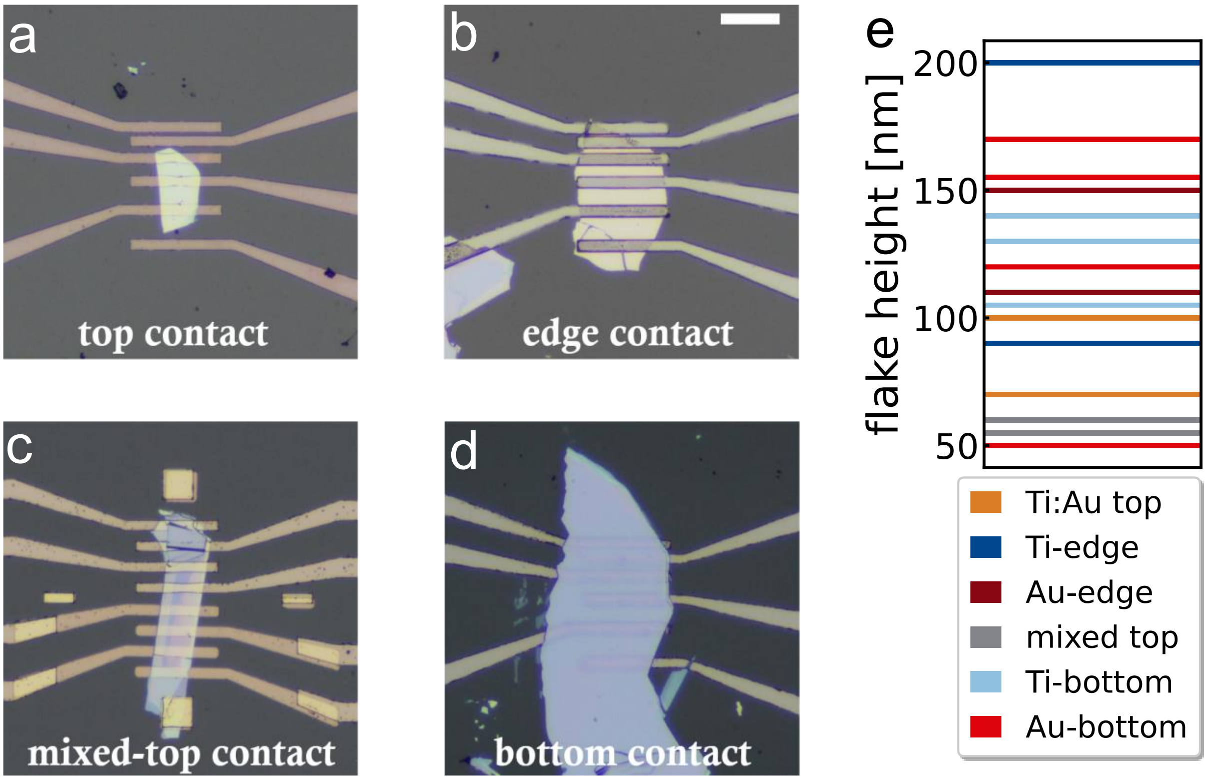


**Figure SI1**. Light microscopy images of $MoS_2$ flakes with different electrode styles: a) edge contact; b) top contact; c) mixed top contact; d) bottom contact. e) Flake height for every examined flake, measured with a profilometer (*Dektak XT*). The thickness ranges from 50 nm to 200 nm. The scale bar is 50 µm.

## SEM-images of an etched flake

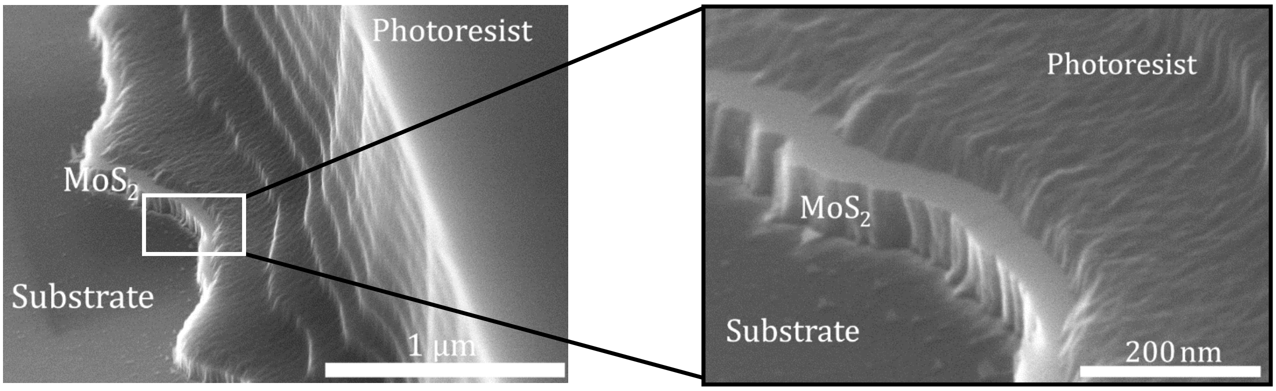


**Figure SI2.** SEM images of an etched $MoS_2$ flake. a) Overview with visible resist flank, b) zoomed in image of the marked spot in a). The height of this particular flake is approximately 70 nm.

## Lithographic process for fabricating edge contacts

For bottom contacts, the lithography is done prior to the exfoliation. For top contacts, the fabrication is exactly the same as in Figure SI4, only the etching part (Figure SI4c) is left out.

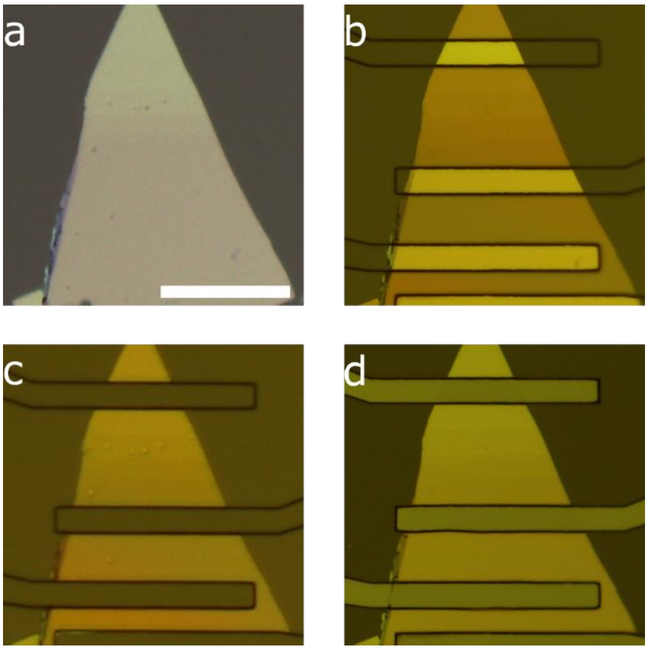


**Figure SI3.** Fabrication process of an edge contact: a) exfoliated flake on a glass substrate; b) patterned electrodes after photolithography and resist development; c) flake after $O_2/SF_6$ plasma etching; d) flake after evaporation of the electrodes. The scale bar is 50 µm.

Edge contacts are obtained by following the lithographic steps depicted in Figure SI3. The mixed top contacts are fabricated in two lithographic processes: In the first one, the upper four electrode fingers, cf. Figure SI1c, are made as well as the leads with 4 nm Ti and 20 nm Au. After lift-off, the lithography is repeated and the lower four electrodes consisting of 25 nm Au are evaporated without a wetting layer of titanium.

## Persistent photocurrents in $MoS_2$ flakes

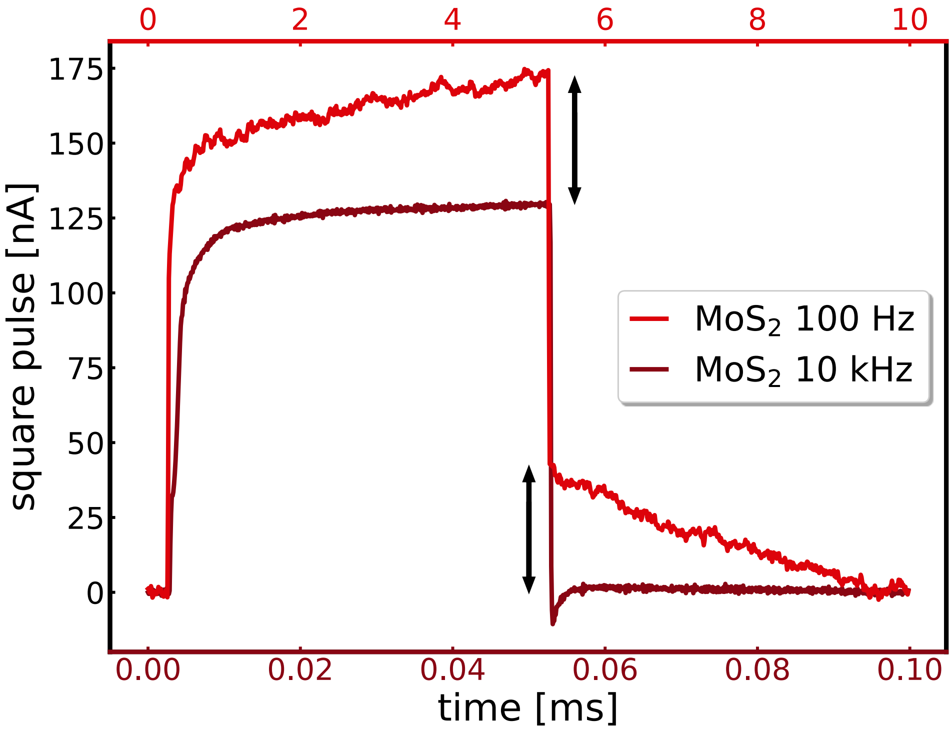

**Figure SI4.** 635 nm square pulse measurements of a 20 µm Au edge contact under 1 V bias. Bright red shows the measurement with a laser repetition rate of 100 Hz and dark red with 10 kHz. The two black arrows are equally long, indicating the height difference caused by the slow parts in the rise and fall time for the 100 Hz measurement.

For measurements at 100 Hz, a persistent photocurrent is sometimes observable, more pronounced for higher voltages. This can be seen in a steep increase and decrease of the rise and fall time respectively, followed by a slow and steady increase / decrease, cf. Figure SI4 bright red curve. These two effects can be attributed to the fast photoconductive and the slower photogating effect. Di Bartolomeo et al.[1] examined that the slower photogating relies on deeper traps which yield very long response times, but require a longer exposure to be filled. Thus, this behaviour can be suppressed when switching to higher repetition rates like 10 kHz, showing only the relevant photoconductive component.

## I-V characteristics

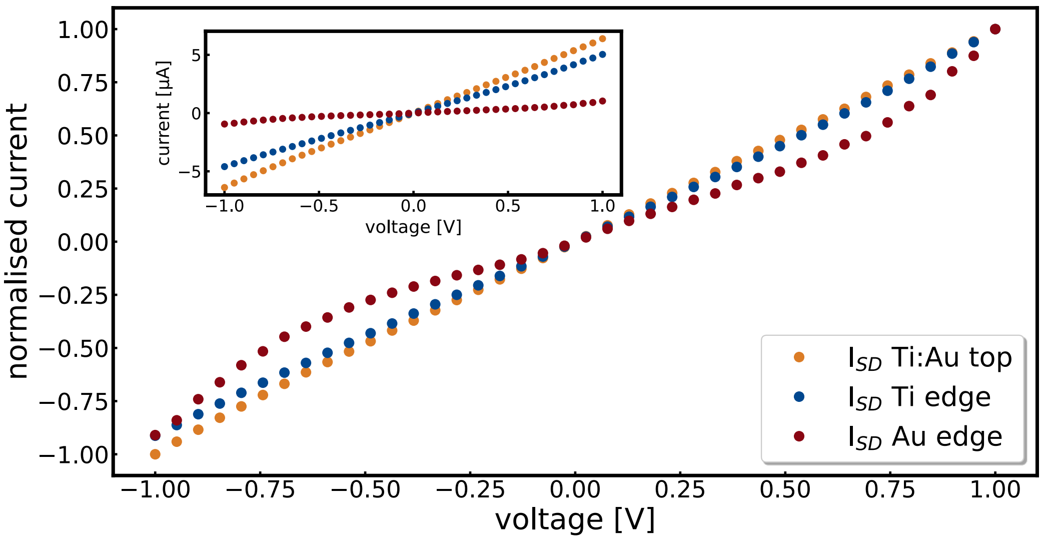


**Figure SI5.** Normalized I-V characteristics of a Ti:Au top (ochre), a Ti edge (blue) and a Au edge (red) contacted flake without illumination. Measurements made on 10 µm contacts from -1 to 1 V. The inset shows the absolute current values. The slope can be translated into resistances of 161 kΩ, 218 kΩ and 1.46 MΩ respectively.

The I-V characteristic shows the same trend observed in literature: for a titanium contacting, either top or edge, there is an (nearly) ohmic behaviour, whereas for a gold contacting the curves tend to differ due to the higher Schottky barrier. The dark currents are generally rather high, due to the bulk $MoS_2$ flakes.

## Band diagram of an Au top and an Au edge contact

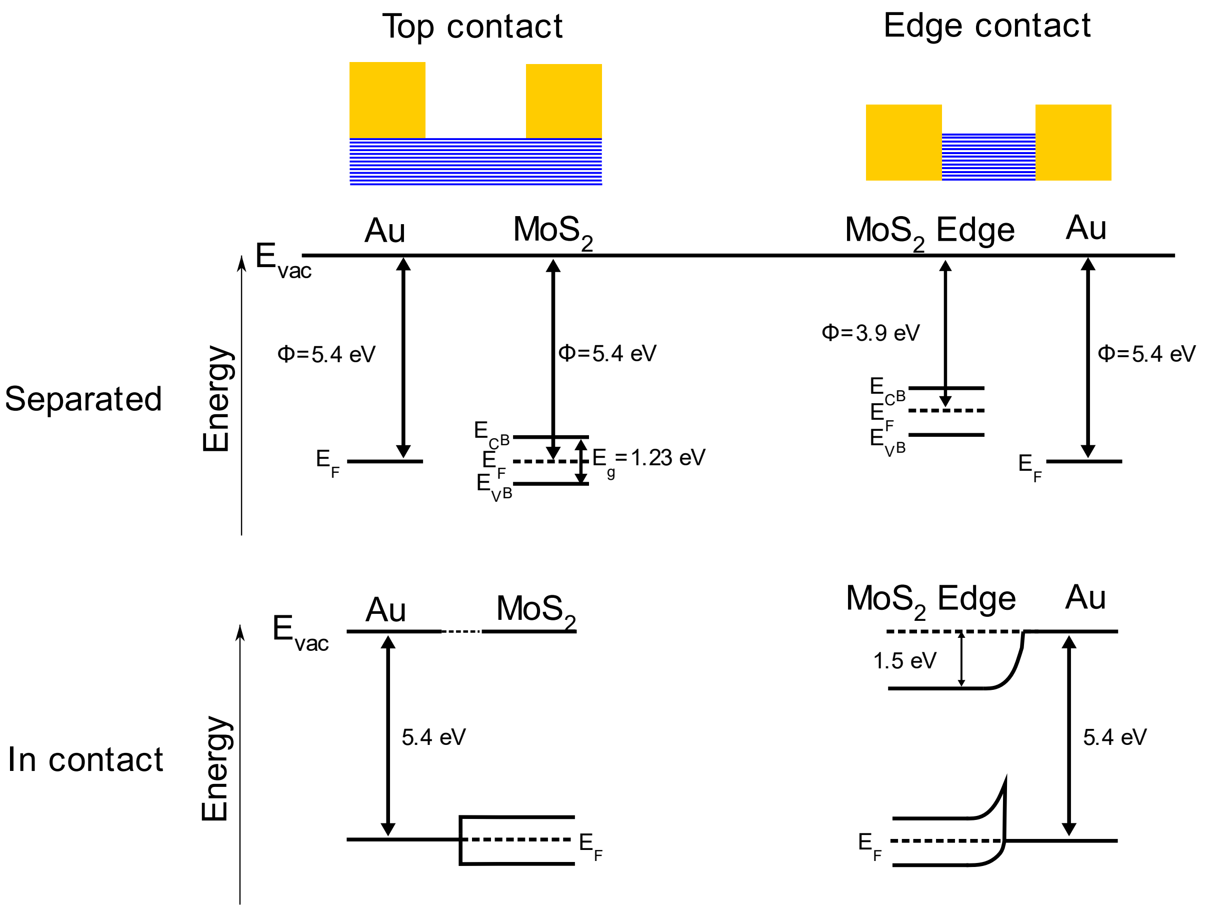


**Figure SI6.** Band diagrams for an idealized top contact (left) and edge contact (right). The upper half shows all the work functions extracted from literature[2–5]. At the bottom, an idealized model of the materials brought into contact can be seen.

The band diagram in Figure SI6 shows an idealized model of a top and an edge contact consisting of gold and $MoS_2$. Thereby, it is important to use the appropriate $MoS_2$ work function, depending on the electrode geometry. When contacting the edge, a larger Schottky barrier arises then when contacting from the top. It is important to emphasise, that those literature values may differ in reality, depending on the nature of the semiconductor (n- or p-type or intrinsic), the exact work function of Au and stress, strain, defects or any order form of imperfection in the $MoS_2$ flake, to name just a few examples. Additionally, metallization at the edge contact might occur, altering the band model[6].

**Rise / fall time vs channel length and voltage**

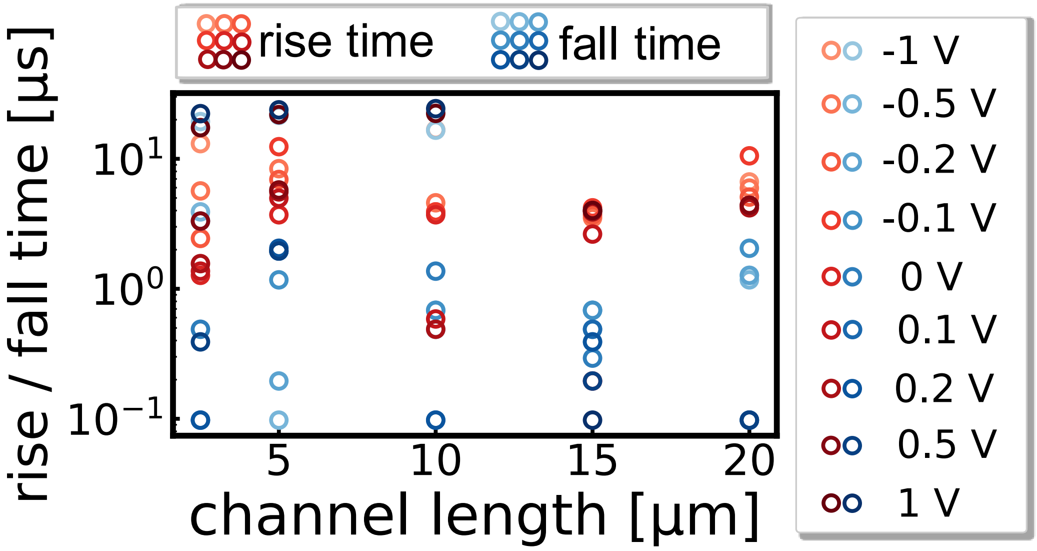


**Figure SI7.** Rise times (red) and fall times (blue) of 10 kHz measurements of an Au edge contact plotted vs the channel length. The different shades resemble the different biases applied.

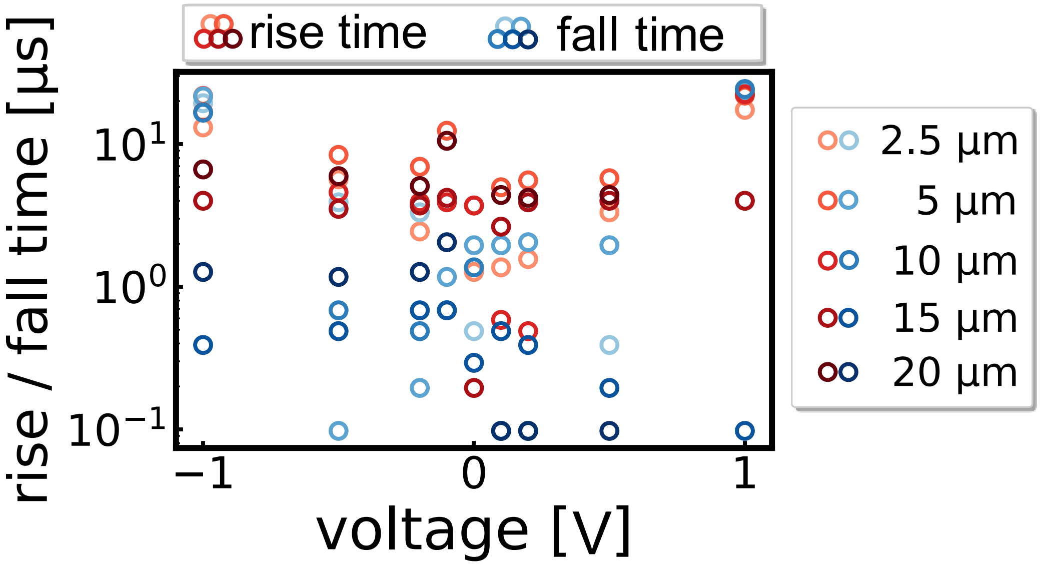


**Figure SI8.** Rise times (red) and fall times (blue) of 10 kHz measurements of an Au edge contact plotted vs the bias voltage. The different shades resemble the different channel lengths of the electrodes.

There is no clear trend visible towards the applied voltage or channel length. Therefore, we cannot further determine the speed limiting mechanism of the $MoS_2$ flakes.

## Impulse response and bandwidth of the fastest device

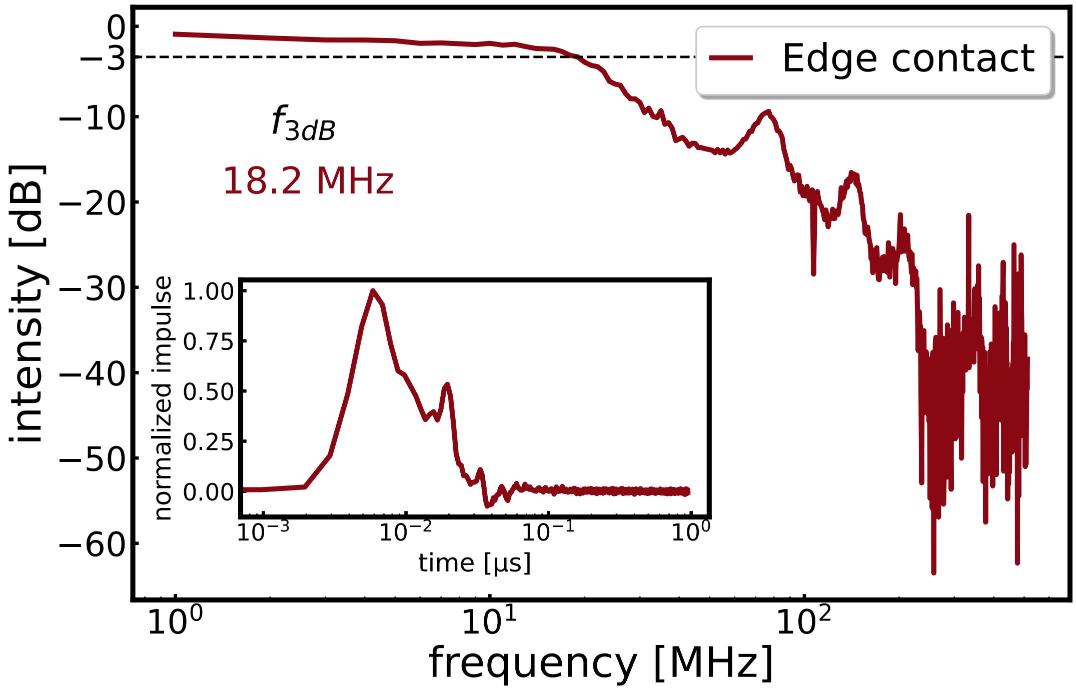


**Figure SI9.** Bandwidth of fastest $MoS_2$ photodetector fabricated. The inset shows the impulse response of a 10 µm channel with 0.5 V applied bias of an Au edge contacted device towards a 636 nm laser operated at 1 MHz repetition rate. The graph shows the Fourier transformation of the impulse response.

## References


(1) di Bartolomeo, A.; Genovese, L.; Foller, T.; Giubileo, F.; Luongo, G.; Croin, L.; Liang, S. J.; Ang, L. K.; Schleberger, M. Electrical Transport and Persistent Photoconductivity in Monolayer $MoS_2$ Phototransistors. *Nanotechnol.* **2017**, *28*, 214002

(2) Asadi, M.; Kumar, B.; Behranginia, A.; Rosen, B. A.; Baskin, A.; Repnin, N.; Pisasale, D.; Phillips, P.; Zhu, W.; Haasch, R.; Klie, R. F.; Král, P.; Abiade, J.; Salehi-Khojin, A. Robust Carbon Dioxide Reduction on Molybdenum Disulphide Edges. *Nat. Comm.* **2014**, *5*.

(3) Greulich, K.; Belser, A.; Bölke, S.; Grüninger, P.; Karstens, R.; Sättele, M. S.; Ovsyannikov, R.; Giangrisostomi, E.; Basova, T. v.; Klyamer, D.; Chassé, T.; Peisert, H. Charge Transfer from Organic Molecules to Molybdenum Disulfide: Influence of the Fluorination of Iron Phthalocyanine. *J. Phys. Chem. C* **2020**, *124*, 16990–16999.

(4) Uda, M.; Nakamura, A.; Yamamoto, T.; Fujirnoto, Y. Work Function of Polycrystalline Ag, Au and Al. *J. Electron Spectros. Relat. Phenomena* **1998**, *91*, 643–648.

(5) Baik, S. S.; Im, S.; Choi, H. J. Work Function Tuning in Two-Dimensional MoS2 Field-Effect-Transistors with Graphene and Titanium Source-Drain Contacts. *Sci Rep* **2017**, *7*, 1–8.

(6) Yang, Z.; Kim, C.; Lee, K. Y.; Lee, M.; Appalakondaiah, S.; Ra, C. H.; Watanabe, K.; Taniguchi, T.; Cho, K.; Hwang, E.; Hone, J.; Yoo, W. J. A Fermi-Level-Pinning-Free 1D Electrical Contact at the Intrinsic 2D $MoS_2$–Metal Junction. *Adv. Mater.* **2019**, *31*.
